# Health-related quality of life and complications of corticosteroid treatment in patients with immune thrombocytopenia in two teaching hospitals in Ethiopia: a cross-sectional study

**DOI:** 10.3389/fmed.2024.1423161

**Published:** 2024-11-05

**Authors:** Dessale Abate Beyene, Eskinder Ayalew Sisay, Atalay Mulu Fentie, Amha Gebremedhin

**Affiliations:** ^1^Tikur Anbessa Specialized Hospital, Department of Pharmacology and Clinical Pharmacy, School of Pharmacy, College of Health Sciences, Addis Ababa University, Addis Ababa, Ethiopia; ^2^Tikur Anbessa Specialized Hospital, School of Medicine, College of Health Sciences, Addis Ababa University, Addis Ababa, Ethiopia

**Keywords:** corticosteroids, Ethiopia, health-related quality of life, immune thrombocytopenia, immune thrombocytopenia life quality index, platelet count

## Abstract

**Background:**

The treatment of immune thrombocytopenia (ITP) is a major clinical challenge and has a significant impact on health-related quality of life (HRQoL), and prolonged use of corticosteroids may have a negative impact on HRQoL.

**Objectives:**

To evaluate the impact of ITP on HRQoL and complications of corticosteroid treatment in patients with ITP in two teaching hospitals in Ethiopia.

**Methods:**

The institution-based cross-sectional study was conducted from November 15, 2022, to March 15, 2023, to recruit 214 study participants during the study period (4 months). The ITP Life Quality Index (ILQI) in the Amharic version was used to assess the impact of ITP. Linear regression analysis models were also used, and a *p*-value of less than 0.05 was generally considered statistically significant.

**Results:**

Regarding treatment, the majority 172 (80.4%) of study participants were taking prednisolone only, and 143 (66.8%) of study participants had at least one side effect of corticosteroids during the entire treatment period. Predictive factors for a higher impact of ITP on HRQoL; all correlated variables explained 36.5% (adjusted R-squared = 0.365, *p* < 0.0001) of the variance and had a moderate impact on HRQoL. Furthermore, predictive factors for an increasingly higher impact of ITP on HRQoL were the development of emotionally related corticosteroid side effects (*β* = 0.392, 95% CI: 5.160–9.961, *p* < 0.001), the presence of fatigue during the assessment (*β* = 0.326, 95% CI: 4.394–9.475, *p* < 0.001), patients not taking cotrimoxazole prophylaxis treatment (*β* = 0.236, 95% CI: 2.236–6.570, p < 0.001), living far from the hematology clinic (outside Addis Ababa) (*β* = 0166, 95% CI: 1.107–5.114 *p* = 0.003), having epistaxis and/or wet purpura (mucosal bleeding) (*β* = 0.191, 95% CI: 0.091–4.259, *p* = 0.001), and skin symptoms (petechiae and ecchymosis) (*β* = 0.041, 95% CI: 0.091–4.259 *p* = 0.041) during diagnosis.

**Conclusion:**

The impact of ITP on their energy levels and work life was high compared to the impact of ITP on daily life. The side effects of corticosteroids also affect the HRQoL of ITP patients.

## 1 Introduction

Immune thrombocytopenia (ITP) is an acquired form of bleeding disorder that results from the destruction of blood platelets by autoantibody-mediated and cell-mediated platelets, leading to accelerated platelet clearance and impaired thrombopoiesis (1–3). It is described as a transient or persistent reduction in platelet count <100 ×10^9^/L and an increased risk of bleeding that depends on the degree of thrombocytopenia (4, 5).

According to the ITP World Impact Survey, petechiae (64%) and bruising of unknown origin (65%) were the most common clinical presentations at diagnosis (6). Many patients experience a range of physical and emotional symptoms due to their medical condition. Research shows that, for a significant portion of patients, maintaining a stable platelet count is a major concern. This is because low platelets can result in bleeding, which can be a serious health risk. Patients frequently report fatigue, anxiety, fear of bleeding, and frustration, which are the most debilitating aspects of ITP patients’ HRQoL concerning their condition (6). These symptoms can significantly impact patients’ HRQoL in various ways, including their ability to perform daily activities, their emotional well-being, their energy levels, their ability to work, and their overall productivity (7–10).

ITP requires lifelong treatment in a significant proportion of adult patients, and its treatment is challenging (11, 12). Treatment outcomes are assessed based on the clinical response rate, treatment relapse rate, platelet count before and after the treatment, HRQoL, and adverse events of the treatment/procedure (4, 13–18). Therefore, treatment should be tailored to the individual patient, considering factors such as age, lifestyle, comorbidities, compliance, patient preferences, the presence and severity of bleeding, and the potential treatment side effects (19). For this reason, the improvement of HRQoL parameters was named as an important treatment goal in the 2019 updated guidelines of the American Society of Hematology (ASH) and the International Consensus Report (13, 20).

In resource-limited countries like Ethiopia, physicians face high patient burden and limited clinic time (21, 22); due to this treating physicians usually prioritize addressing low platelet counts and avoiding life-threatening bleeds over HRQoL. Patients with ITP are mainly concerned with how the disease affects their daily lives, including their overall well-being and ability to function (12, 23). Prolonged use of corticosteroids in adults may have a negative impact on HRQoL due to the effects on sleep disturbances, weight gain, and mental health (24). Patient-reported HRQoL has proven to be an important tool for understanding ITP and its treatments from the patient’s perspective. The information provided by the patients is valuable as it offers insights into their experiences, which have been underrepresented in clinical research so far (25, 26).

Several studies have assessed the HRQoL of ITP patients using different instruments, such as the 36-item Short-Form Health Survey, the EuroQol Questionnaire with five dimensions, the ITP Patient Assessment Questionnaire (ITP-PAQ), and the ITP Life Quality Index (ILQI) (9, 10, 27–29). The more recently developed instrument for assessing HRQoL in ITP patients was the ILQI tool. The content and psychometric validity of the ILQI were established in 14 countries (the United States, China, the United Kingdom, France, Germany, Italy, India, Canada, Turkey, Japan, Colombia, Spain, Egypt, and Ethiopia) (30, 31).

To our knowledge, there is still no published evidence-based literature performed in Ethiopia that assesses the impact of ITP on the HRQoL of ITP patients. The cornerstone of most ITP treatments was corticosteroid common side effects, and the impact on HRQoL was not assessed. Hence, this study aimed to investigate the impact of ITP on the HRQoL of ITP patients and to determine the factors associated with the HRQoL of ITP patients in Tikur Anbessa Specialized Hospital (TASH) and St. Paul’s Hospital Millennium Medical College (SPHMMC).

## 2 Materials and methods

### 2.1 Study setting

This study was conducted at TASH and SPHMMC, the outpatient departments of both hospitals care for patients in their various specialist clinics, with the hematology clinic being the largest. According to data from the Health Management Information System (HMIS) of the TASH and the SPHMMC, an average of 50 ITP and 20 ITP patients, respectively, visit the hematology clinic each month.

### 2.2 Study design and period

This institution-based cross-sectional study was conducted to evaluate the impact of ITP on the HRQoL of ITP patients who were followed up in the hematology clinic of TASH and SPHMMC during the study period from November 15, 2022, to March 15, 2023.

### 2.3 Eligibility criteria

Patients attending the hematology outpatient clinics of both hospitals during the study period and who had a confirmed diagnosis of ITP according to the 2019 ASH guidelines and the standardization of terminology, definitions, and outcome criteria in ITP in adults and children (primary, secondary, newly diagnosed, persistent, chronic and severe ITP) (4, 20), patients aged ≥14 years and patients who were willing to participate in the study were included. Patients who had not started treatment and had incomplete medical records were excluded. Secondary ITP patients with active disease (not clinically stable) due to their primary condition were also excluded.

### 2.4 Sample size determination and sampling technique

All ITP patients who visited TASH and SPHMMC during the study period (4 months) were recruited. Due to the rarity of the incident, all patients who received ITP treatment during the study period and met the eligibility criteria were included in the study. Study participants were recruited from TASH and SPHMMC using a consecutive sampling technique.

### 2.5 Data collection and management

#### 2.5.1 Data collection instruments

##### 2.5.1.1 Data abstraction form

The data abstraction format is a useful tool designed to extract information from either the medical record or directly from the patient. This information includes sociodemographic characteristics such as age, sex, educational status, place of residence, marital status, and healthcare costs. Additionally, it helps in gathering clinical characteristics such as the type of ITP, duration of symptoms, comorbidity, physical and clinical presentations at diagnosis, and presence of bleeding. Furthermore, the data abstraction format assists in collecting treatment-related characteristics such as the type, frequency, and duration of treatment. After conducting an extensive literature review (17, 24, 25, 32) and with the help of experts, structured questionnaires were designed to evaluate the side effects of corticosteroid treatment in patients with ITP.

##### 2.5.1.2 ITP life quality index (ILQI)

The ILQI is a 10-item patient-reported outcome measure developed to aid discussions between patients and physicians about the patient’s disease experience over the last month (30). The ITP World Impact Survey utilized the ILQI tool to collect real-world data about the challenges, experiences, and perspectives of ITP patients (33). The responses range from ‘never’ to ‘always’. Three of the questions (1, 2, and 5) have additional response options that allow patients to indicate if the question is not relevant or if they do not wish to answer. If the patient selects ‘I am not currently working/studying due to ITP’, the value 4 applies, and if they select ‘I am not currently working/studying due to other reasons or does not apply/prefer not to say’, the value 0 applies. A total sum score between 7 and 40 was originally proposed, where a lower score indicates a lower impact and a higher score indicates a higher impact of ITP on HRQoL. The content and psychometric validity of the ILQI was assessed in 13 countries (30). The acceptability, reliability, and validity of the psychometric properties of the Amharic version were also assessed and validated in the Ethiopian population (31).

### 2.6 Data quality assurance and outcome measurements

The validated Amharic version of the ILQI was used to assess the HRQoL of patients treated for ITP (31). A pretest was then administered to 5% of ITP patients. The purpose of the pretest was to ensure that respondents understood the questions and could review the wording, logic, and skip order in a way that made sense to respondents. Based on the results of the pretest, appropriate corrections were made before the actual study was conducted. Data collectors were recruited by two clinical pharmacists and one nurse, and a half-day training was given by the principal investigator about the objectives of the study and how to use the tool to collect data directly from the patient and medical records/charts. To ensure completeness and consistency of the data, all data were reviewed daily by the principal investigator to ensure the quality of the data.

HRQoL scores were calculated using the ILQI tool. According to the instructions for use, raw scores for the ranges of ILQI were calculated and transformed to a scale of 0 to 100 by using the formula for mean transformation.


Domainx¯=100MAXs−Nx∑x¯−N

Where Domain *x̄*: domain mean transformed to 100; MAXs: maximum score; *N*: number of items; ∑ *x̄*: sum of means; 100 is the highest impact of ITP on HRQoL, and 0 is the lowest impact of ITP on HRQoL.

### 2.7 Data analysis

Data was entered and analyzed using SPSS version 26. Descriptive statistics, such as frequency, median, and range, were utilized to summarize sociodemographic data, as well as clinical and treatment characteristics. After checking the assumptions, univariate analysis was performed to obtain candidate variables for the multivariate regression model to determine possible predictors of the HRQoL variables. In the univariate analysis, factors associated with HRQoL showed a marginal association at *p* < 0.2 after univariate analysis, and all clinically relevant variables were considered candidate variables for the multivariate linear regression model to identify strong factors associated with HRQoL, respectively. A *p*-value <0.05 was considered to indicate statistical significance.

## 3 Results

### 3.1 Sociodemographic characteristics of the study participants

A total of 214 study participants took part in this study; the majority 153 (71.5%) of them were from TASH. Most 161 (75.5%) were female patients. Regarding the age distribution, the median age of the study participants was 30 years and ranged from 15 to 88 years, and most 78 (36.4%) participants were in the 25–34 years age group. One-third of the study participants had a university degree or more 76 (35.5%) and were also self-employed 59 (27.6%). Most participants 197 (92.1%) lived with their family, and half of them 109 (50.9%) lived far from the hematology clinic (outside Addis Ababa). In addition, more than half of the health care costs of the study participants 133 (62.1%) were borne by the patients themselves or by their relatives (Table 1).

**Table 1:** Sociodemographic characteristics of ITP patients attending the TASH and SPHMMC hematology clinics in Addis Ababa, Ethiopia, 2022 (*n* = 214).

Variables		Frequency	Percentage
Study site	TASH	153	71.5
	SPHMMC	61	28.5
Sex	Female	161	75.2
	Male	53	24.8
Age	14–24	58	27.1
	25–34	78	36.5
	35–44	40	18.7
	45–54	20	9.3
	55 and above	18	8.4
Marital status	Married	149	69.6
	Never married	58	27.1
	Widowed	4	1.9
	Divorced	3	1.4
Educational level	Unable to read and write	7	3.3
	Enable read and write	12	5.6
	Primary education (grades 1–8)	33	15.4
	Secondary Education (grades 9–12)	54	25.2
	Diploma/Certificate	32	15.0
	Degree and above	76	35.5
Occupational status	Employed	59	27.6
	Housewife	47	22.0
	Self-Employed	45	21.0
	Student	41	19.2
	Unemployed	20	9.3
	Retired	2	0.9
Residence	Outside Addis Ababa	109	50.9
	Addis Ababa	105	49.1
Health service charge	With cash	133	62.1
	With health insurance	56	26.2
	With free	25	11.7
With whom do you live?	With family	197	92.1
	Alone	17	7.9

TASH, Tikur Anbessa Specialized Hospital; SPHMMC, St. Paul’s Hospital Millennium Medical College.

### 3.2 Clinical characteristics of ITP patients during diagnosis

**Figure 1:**
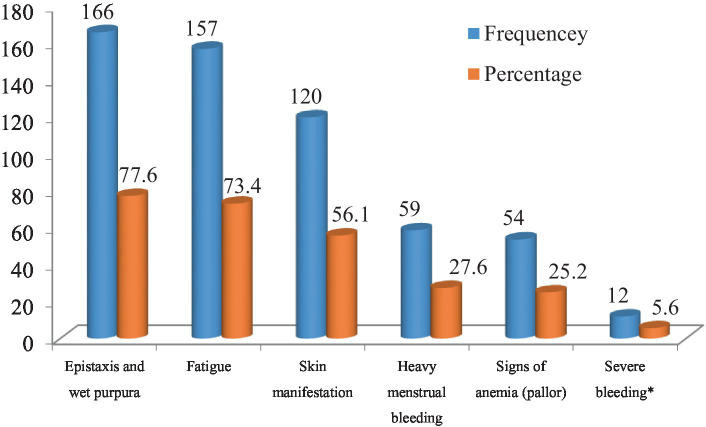
Clinical presentations of ITP patients during diagnosis attending the TASH and SPHMMC hematology clinics in Addis Ababa, Ethiopia, 2022 (*n* = 214). Severe bleeding*; gastrointestinal bleeding, Intracranial bleeding, rectal bleeding, retinal hemorrhage.

As shown in Figure 1, 166 (77.6%) of the study participants had epistaxis and/or wet purpura (mucosal bleeding), 157 (73.4%) had fatigue, 120 (56.1%) had skin manifestations (petechiae, purpura, ecchymosis) during diagnosis. In addition, 59 (36.6%) of the women had heavy menstrual bleeding (both in terms of volume and duration), 54 (25.5%) of the study participants also had signs of anemia (pallor) and only 12 (5.6%) had severe bleeding (gastrointestinal bleeding, intracranial bleeding, rectal bleeding and retinal bleeding).

**Figure 2:**
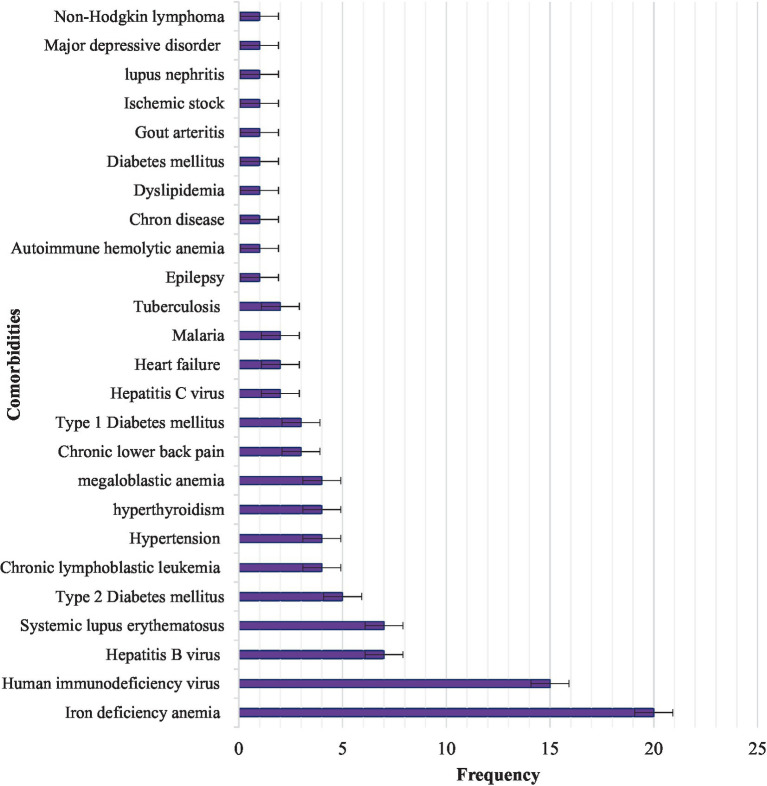
Comorbidities in ITP patients attending the TASH and SPHMMC hematology clinics in Addis Ababa, Ethiopia, 2022 (*n* = 214).

In this study, 91 (42.5%) participants had comorbidities and the most common comorbidity was iron deficiency anemia, which was secondary to bleeding (Figure 2) The median age of study participants at diagnosis of ITP was 27 years (ranging from 9 to 86 years), the median duration of ITP since diagnosis was 24 months (ranging from 3 to 240 months). During the assessment, the most common symptom of ITP was fatigue 53 (25.2%), followed by headache 14 (6.5%) (Table 2).

**Table 2:** Clinical characteristics of ITP patients attending the TASH and SPHMMC hematology clinics in Addis Ababa, Ethiopia, 2022 (*n* = 214).

Variables		Frequency	Percentage
Comorbidity	Yes	91	42.5
	No	123	57.5
Current clinical presentations of ITP	Fatigue	54	25.2
	Headache	14	6.5
	Depression	8	3.7
	Weight loss	3	1.4
	Bleeding	1	0.5
The Median (range) of age	Age at Diagnosis in a year	27 (9–86) years	
	Duration since ITP diagnosis in a month	24 (3–240) months	

Iron deficiency anemia 20 (22.0%), followed by HIV 15 (16.5%), hepatitis B virus (HBV) 7 (7.7%), and systemic lupus erythematosus (SLE) 7 (7.7%), accounted for the highest proportion of comorbidities in ITP patients attending TASH and SPHMMC during the study period (Figure 2).

### 3.3 Treatment-related characteristics of ITP

For the treatment of ITP, most of the study participants 172 (80.4%) were prescribed prednisolone alone, followed by combinations of prednisolone and dexamethasone 31 (14.5%) as first-line treatment in this study setting. [Azathioprine or rituximab] + prednisolone 20 (36.4%) were used as second-line treatment options for ITP.

Approximately 63 (29.4%) of the study participants received platelet transfusions to prevent bleeding, and 27 (12.6%) tranexamic acid to stop bleeding. In addition, approximately 121 (56.5%), 100 (46.7%), and 45 (21.0%) study participants were prescribed cotrimoxazole prophylaxis (CPT), proton pump inhibitors (PPI), and calcium with vitamin D3 supplement as prophylaxis to prevent immunosuppression-related infections, peptic ulcers, and osteoporosis, respectively (Table 3).

**Table 3:** Treatment-related characteristics of ITP patients attending the TASH and SPHMMC hematology clinics in Addis Ababa, Ethiopia, 2022 (*n* = 214).

Variables	Frequency	Percentage
First-line treatment of ITP		
Prednisolone alone	172	80.4
Prednisolone + Dexamethasone	31	14.5
Dexamethasone alone	6	2.8
Prednisolone + Methylprednisolone	5	2.3
Second-line treatments of ITP		
[Azathioprine or Rituximab] + Prednisolone	20	36.4
[Rituximab alone] or [Prednisolone alone] or [Azathioprine alone]	10	18.2
Rituximab + Splenectomy + [Azathioprine or Prednisolone]	8	14.5
[Rituximab + Azathioprine] ± Prednisolone	7	12.7
[Splenectomy + Prednisolone] or [Splenectomy + Rituximab]	6	10.9
[Splenectomy + Azathioprine+ Prednisolone] ± Rituximab	4	7.3
Other medications to stop bleeding		
Platelet transfusion	63	29.4
Tranexamic acid	27	12.6
For prophylaxis of corticosteroid Complications		
Cotrimoxazole prophylaxis treatment	121	56.5
Proton pump inhibitors	100	46.7
Calcium with Vitamin D3 supplementation	45	21.0

### 3.4 Corticosteroid side effects in ITP patients

**Figure 3:**
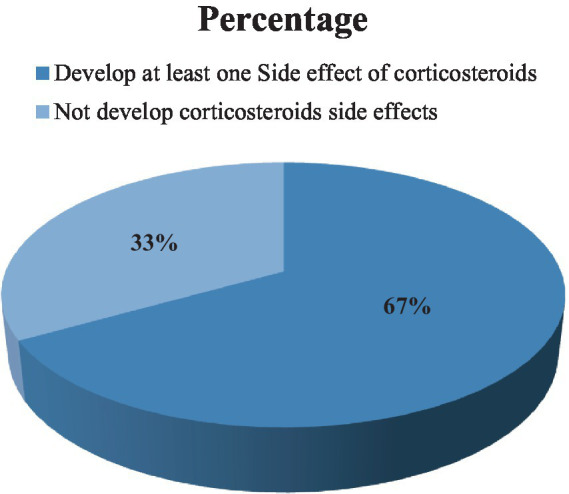
Percentages of corticosteroid side effects in ITP patients attending the TASH and SPHMMC hematology clinics in Addis Ababa, Ethiopia, 2022 (*n* = 214).

In this study, 143 (66.8%) of the study participants experienced at least one side effect of corticosteroids during the entire treatment period, such as physical appearance, emotional corticosteroids, and physical symptoms (Figure 3).

Regarding the physical appearance-related side effects, weight gain/increased appetite 86 (40.2%), followed by a moon face, bloating, and swelling 72 (33.6%) accounted for the highest proportion. Among emotional corticosteroid side effects, insomnia, restlessness, and/or sleep disturbances 70 (32.7%) and physical symptoms related to corticosteroid side effects, general weakness/fatigue 102 (47.7%), and muscle weakness 30 (14.0%) accounted for the largest proportion. On the other hand, corticosteroid-related complications, such as increased blood glucose 19 (8.9%), increased blood pressure 15 (7.0%), iatrogenic Cushing’s syndrome 9 (4.2%), and osteoporosis 5 (2.3%), occurred in the study participants (Table 4).

**Table 4:** Corticosteroid side effects in ITP patients attending the TASH and SPHMMC hematology clinics in Addis Ababa, Ethiopia, 2022 (*n* = 214).

Variables	Frequency	Percentage
Physical appearance-related corticosteroid side effects		
Weight gain / increased appetite	86	40.2
Moon face, bloating, swelling	72	33.6
Stretch mark	11	5.1
Acne	7	3.3
Hair loss	6	2.8
Emotional-related corticosteroid side effects		
Insomnia, restlessness, and/or trouble sleeping	70	32.7
Depression and/or stress	17	7.9
Anxiety and/or nervousness	12	5.6
Anger and/or irritability	8	3.7
Physical symptoms related to corticosteroid side effects		
Generalized weakness, fatigue	102	47.7
Muscle weakness	30	14.0
Visual problems (light sensitivity/ decreased visual acuity)	12	5.6
Dizziness, headaches	34	15.9
Nausea, upset stomach, vomiting, diarrhea	10	4.7
Other corticosteroid-related complications/Side effects		
Increase blood glucose	19	8.9
Increase blood pressure	15	7.0
Iatrogenic Cushing’s syndrome	9	4.2
Osteoporosis	5	2.3

### 3.5 Mean scores for the impact of ITP on health-related quality of life

The impact of ITP on their energy levels accounts for the highest mean value (2.53 ± 1.17), followed by the impact of ITP on their working lives or studies (2.51 ± 1.10). On the other hand, the impact of ITP on their sex life (1.04 ± 0.71) was lower than other parameters in the IQLI tool (Table 5).

**Table 5:** Mean scores for the impact of ITP patients attending the TASH and SPHMMC hematology clinics in Addis Ababa, Ethiopia, 2022 (*n* = 214).

Item	ITP life quality index tool	Mean ± SD
Item-1	How often has your ITP impacted your working life or studies?	2.51 ± 1.10
Item-2	How often have you taken me off work or education because of your ITP?	2.49 ± 1.11
Item-3	How often has your ITP impacted your ability to concentrate on everyday tasks?	2.00 ± 1.10
Item-4	How often has your ITP impacted your social life?	1.86 ± 1.08
Item-5	How often have your ITP impacted your sex life?	1.04 ± 0.71
Item-6	How often have your ITP impacted your energy levels?	2.53 ± 1.17
Item-7	How often has your ITP impacted your undertaking of daily tasks?	2.00 ± 1.27
Item-8	How often has your ITP impacted your ability to support people close to you?	1.88 ± 1.14
Item-9	How often has your ITP negatively impacted your hobbies?	1.87 ± 1.12
Item-10	How often has your ITP negatively impacted your normal capacity to exercise?	2.48 ± 1.15

The overall mean score for the impact of ITP on HRQoL according to the IQLI tool was 35.41 ± 9.27. The mean score for the impact of ITP on their work or study was 50.01 ± 2.17, and the impact of ITP on daily life was 31.69 ± 7.38 (Table 6).

**Table 6:** Domain-transformed mean score of ITP patients attending the TASH and SPHMMC hematology clinics in Addis Ababa, Ethiopia, 2022 (*n* = 214).

Variables	Likert scale Mean ± SD	The formula for domain mean*	Domain mean of 100
Impact of ITP on work or study (Items 1 and 2)	2.50 ± 1.09	100/ (8-2) × (5.00–2)	50.01 ± 2.17
Impact of ITP on daily live (Item 3 to Item 10)	1.95 ± 0.92	100/ (32-8) × (15.6–8)	31.69 ± 7.38
The overall mean score	2.06 ± 0.93	100/ (40-10) × (20.64–10)	35.41 ± 9.27

*Domain mean transformed to 100 = 100/(maximum score – no of items) × (sum of means – no of items).

### 3.6 Association between health-related quality of life of ITP patients and explanatory variables

A one-way ANOVA was performed to compare the effects of sociodemographic and clinical characteristics and the corticosteroid side effect variables on HRQoL, and the results of the comparative statistical analysis of the mean values of the HRQoL IQLI domains as a function of the categorical sociodemographic and clinical characteristics and the corticosteroid side effect variables are shown in Table 7. Patients who had skin manifestations (petechiae and ecchymosis) and epistaxis and/or wet purpura (mucous membrane bleeding) during diagnosis (*p* < 0.0001), patients not taking CPT (*p* < 0.0001), patients taking PPI (*p* = 0.007) and calcium with vitamin D3 supplementation (*p* = 0.034), patients who developed iatrogenic Cushing’s syndrome (*p* = 0.02), physical appearance, emotional symptoms, and physical symptoms related to corticosteroid side effects (*p* < 0.0001), patients who had fatigue during the assessment (*p* < 0.0001) and patients living far from the hematology clinic (outside Addis Ababa) (*p* = 0.038) had statistically significant associations with the higher impact of ITP on HRQoL.

**Table 7:** Comparative statistical analysis of IQLI domain mean scores among patients treated for ITP at TASH and SPHMMC, according to the categorical sociodemographic, clinical characteristics, treatment-related and corticosteroid-related side effects.

	Variables, Mean ± SD		HRQoL	Work	Daily live
1	Place of residence	Addis Ababa	19.3 ± 8.9	4.7 ± 2.2	14.6 ± 7.1
		Out of Addis Ababa	21.9 ± 9.5	5.3 ± 2.2	16.7 ± 7.5
		*p* value	0.038	0.083	0.036
2	Fatigue during assessment	No	18.5 ± 8.2	4.7 ± 2.0	13.9 ± 6.4
		Yes	26.8 ± 9.6	6.0 ± 2.4	20.9 ± 7.6
		*p* value	<0.0001	<0.0001	<0.0001
3	Headache during assessment	No	20.1 ± 9.1	4.9 ± 2.1	15.2 ± 7.2
		Yes	29.0 ± 7.9	6.5 ± 2.3	22.5 ± 6.0
		*p* value	<0.0001	0.007	<0.0001
4	Skin manifestation (petechiae and Ecchymosis)	No	17.9 ± 8.3	4.4 ± 2.1	13.5 ± 6.5
		Yes	22.8 ± 9.5	5.5 ± 2.1	17.3 ± 7.6
		*p* value	<0.0001	<0.0001	<0.0001
5	Epistaxis and wet purpura (mucous membrane bleeding)	No	16.1 ± 6.8	4.0 ± 2.0	12.1 ± 5.1
		Yes	21.9 ± 9.5	5.3 ± 2.1	16.7 ± 7.6
		*p* value	<0.0001	<0.0001	<0.0001
6	Cotrimoxazole prophylaxis treatment (CPT)	No	22.9 ± 9.3	5.6 ± 2.0	17.3 ± 7.5
		Yes	17.8 ± 8.4	4.2 ± 2.1	13.5 ± 6.7
		*p* value	<0.0001	<0.0001	<0.0001
7	Proton pump inhibitors (PPI)	No	22.5 ± 9.7	5.5 ± 2.0	16.9 ± 7.9
		Yes	19.0 ± 8.6	4.6 ± 2.2	14.5 ± 6.7
		*p* value	0.007	0.002	0.013
8	Calcium with Vitamin D3 supplementation	No	19.9 ± 9.1	4.8 ± 2.1	15.1 ± 7.2
		Yes	23.2 ± 9.6	5.8 ± 2.1	17.5 ± 7.8
		*p* value	0.034	0.009	0.058
9	Iatrogenic Cushing’s syndrome	No	20.33 ± 9.1	4.9 ± 2.2	15.4 ± 7.3
		Yes	27.67 ± 9.3	6.4 ± 2.2	21.2 ± 7.9
		*p* value	0.02	0.042	0.02
10	Physical appearance-related corticosteroid side effects	No	17.5 ± 7.5	4.4 ± 2.0	13.2 ± 5.8
		Yes	23.6 ± 9.9	5.6 ± 2.1	18.0 ± 8.0
		*p* value	<0.0001	<0.0001	<0.0001
11	Emotional-related corticosteroid side effects	No	17.9 ± 8.3	4.4 ± 2.1	13.6 ± 6.6
		Yes	25.5 ± 8.9	6.2 ± 1.8	19.3 ± 7.3
		*p* value	<0.0001	<0.0001	<0.0001
12	Physical symptoms related to corticosteroid side effects	No	17.2 ± 7.8	4.2 ± 2.0	12.9 ± 61
		Yes	23.3 ± 9.5	5.6 ± 2.1	17.7 ± 7.6
		*p* value	<0.0001	<0.0001	<0.0001

HRQoL, health-related quality of life, SD: standard deviation.

### 3.7 Factors affecting health-related quality of life in ITP patients

#### 3.7.1 Univariate analysis

In the univariate analysis, 13 of the variables examined showed an association with HRQoL measured by the ILQI. Of these candidate variables, all were categorical variables, 12 of which were binary variables (place of residence, fatigue during the assessment, headache during the assessment, epistaxis and/or wet purpura (mucosal bleeding), skin manifestations (petechiae and ecchymosis), CPT, PPI, calcium with vitamin D3 supplementation, iatrogenic Cushing’s syndrome, physical appearance-related corticosteroid side effects, emotional-related corticosteroid side effects, and physical symptoms related to corticosteroid side effects); the rest were multi categorical variables (education level).

#### 3.7.2 Multivariate linear regression analysis

Of the 13 variables used for multivariate linear regression analysis, six variables were identified as correlated with HRQoL by stepwise and forward multivariate linear regression methods and cross-validated by the hierarchical regression method. When the number of patients experiencing emotional-related corticosteroid side effects increased by one, the impact of ITP on patients’ HRQoL increased by 0.392 (*β* = 0.392, 95% CI: 5.160–9.961, *p* < 0.001). The number of patients with fatigue during assessment increased by one, and the impact of ITP on patients’ HRQoL increased by 0.236 (*β* = 0.326, 95% CI: 4.394–9.475, *p* < 0.001). In patients who did not take CPT, the impact of ITP on patients’ HRQoL increased by 0.236 (*β* = 0.236, 95% CI: 2.236–6.570, *p* < 0.001). In addition, the number of patients with epistaxis and/or wet purpura (mucosal bleeding) increased by one during diagnosis, and the impact of ITP on patients’ HRQoL increased by 0.191 (*β* = 0.191, 95% CI: 0.091–4.259, *p* = 0.001). The number of patients living far from the hematology clinic (outside Addis Ababa) increased by one, the impact of ITP on patients’ HRQoL increased by 0.166 (*β* = 0166, 95% CI: 1.107–5.114 *p* = 0.003), and the number of patients with clinical presentations of skin symptoms (petechiae and ecchymosis) of ITP patients increased by 0.041 (*β* = 0.041, 95% CI: 0.091–4.259 *p* = 0.041).

All correlated variables explained 36.5% (adjusted R-squared = 0.365, *p* < 0.0001) of the variance and had a moderate influence on the dependent variable (HRQoL). Of these, 15.4% of the variance (adjusted R-squared = 0.154, *β* = 0.392, *p* < 0.0001) was accounted for by emotion-related corticosteroid side effects. The tolerance of all independent variables ranged from 0.848 to 1. Thus, there were no multicollinearity problems in the models because all were above 0.2. All standardized residuals in the models were normally distributed (*p* < 0.05), meeting the assumptions of the linear regression model (Table 8).

**Table 8:** Factors associated with the HRQoL of ITP patients attending the TASH and SPHMMC hematology clinics in Addis Ababa, Ethiopia, 2022 (*n* = 214).

Model	Predictor variables	R-square	Adjusted R square	R square change	Change statistics			*p*- value	Predictor variables	*β* (95% CI)	SE
					*F*-change	Df1	Df2				
1	Emotional-related corticosteroid side effects	0.154	0.154	0.154	38.549	1	212	0.000	Emotional-related corticosteroid (ref: No)	0.392 (5.160–9.961)	1.218
2	Fatigue during assessment	0.256	0.249	0.102	28.949	1	211	0.000	Fatigue during assessment (ref:No)	0.326 (4.394–9.475)	1.289
3	CPT	0.309	0.299	0.053	16.038	1	210	0.000	CPT (ref: No)	0.236 (2.236–6.570)	1.099
4	Epistaxis and wet purpura	0.343	0.331	0.034	10.951	1	209	0.001	Epistaxis and wet purpura (ref: No)	0.191 (0.091–4.259)	1.281
5	Residence	0.370	0.355	0.027	9.027	1	208	0.003	Residence (ref: Addis Ababa)	0.166 (1.107–5.114)	1.024
6	Skin manifestation	0.383	0.365	0.013	4.236	1	207	0.041	Skin manifestation (ref: No)	0.041 (0.091–4.259)	1.057

CPT, Cotrimoxazole prophylaxis treatment; *β*, Beta coefficient; SE, standard error; DF, degree of freedom; CI, confidence interval.

## 4 Discussion

This study aimed to investigate the HRQoL of patients with ITP and the factors associated with HRQoL. The updated international consensus report indicates that there are differences in the clinical presentation of ITP, clinical outcomes, and response to treatment (20). Therefore, the assessment of HRQoL, complications of corticosteroid treatment, and the impact of corticosteroid side effects on the HRQoL of ITP patients plays an important role in the quality of outcomes and measuring the success of treatment. The most common clinical presentation at diagnosis of ITP was epistaxis and/or wet purpura (mucosal bleeding) (77.6%), followed by fatigue (73.4%), skin manifestations (petechiae, purpura, and ecchymosis) (56.1%), and (36.6%) of the women had heavy menstrual bleeding (both in terms of volume and duration). This was similar to studies conducted in Mexico (6, 34), in Spain (35), in France (36), and in Turkey (37).

Corticosteroid side effects may affect HRQoL or treatment response, and in this study population, 71.5% of study participants had chronic ITP. There is a significant association between the duration of corticosteroid treatment and the average number of adverse events experienced (24). Emotional corticosteroid side effects, such as insomnia, restlessness, and/or sleep disturbances (32.7%), physical symptoms, including general weakness/fatigue (47.7%), and muscle weakness (14.0%), accounted for the largest proportion. In a study conducted in the United States, patients treated with corticosteroids reported that the number and severity of corticosteroid side effects increased significantly when the treatment duration was extended from 3 months to 12 months (24). According to the ITP World Impact Survey, approximately 50% of study participants suffered from fatigue during the survey (6), and in this study, general weakness/fatigue (47.7%) was also the most common corticosteroid side effect in ITP patients. This can severely affect HRQoL domains, such as energy levels to perform activities. On the other hand, 56.5, 46.7, and 21.0% of the study participants took CPT, PPI, and calcium with vitamin D3, respectively. This supportive treatment reduced the immunosuppression and gastrointestinal side effects of corticosteroids as well as the extent of osteoporosis. In general, to decrease corticosteroid-related side effects, prednisone should be rapidly tapered and usually stopped in responders, and non-responders stop the medications after 4 weeks of initiation (38).

In this study, the total impact of ITP on HRQoL is 35.41 ± 9.27, and the impact of ITP on work or study is 50.01 ± 2.17, which is greater than the impact of ITP on daily life (31.69 ± 7.38); this is consistent with the ITP World Impact Survey data (6, 33) and survey data reported from Switzerland, Austria, and Belgium (39). Our study is also similar to the studies conducted in China (40), the United States of America (9), and Serbia (7), in which physical function was more impaired than in the other domains of HRQoL. On the other hand, an Indian study showed that the impact of ITP on patients’ work/study was less than the impact of ITP on their daily lives (41). A systematic review conducted in 2018 suggests that patients with ITP experience negative effects on their sexual activities, including decreased libido and bruising and bleeding during intercourse (17). In this study, the mean impact of ITP on patients’ sex life was rated as 1.04 ± 0.71, which is lower than all other IQLI domains, and the result is similar to a qualitative study in the United Kingdom, which found that the impact of ITP on sex life was less relevant (42).

Predictive factors for a higher impact of ITP on HRQoL included emotional-related corticosteroid side effects, fatigue during the assessment, not taking CPT, epistaxis and/or wet purpura (mucosal bleeding), place of residence, and skin symptoms (petechiae and ecchymosis). These were significantly correlated with a greater impact of ITP on HRQoL, with emotion-related corticosteroid side effects accounting for the highest value with 15.4% of the variance (adjusted R-squared = 0.154, *β* = 0.392). When the number of patients experiencing emotional-related corticosteroid side effects increased by one, the impact of ITP on patients’ HRQoL increased by 0.392 (*β* = 0.392, 95% CI: 5.160–9.961, *p* < 0.001). This might be due to the emotional side effects of steroids, such as insomnia, depression, anxiety, restlessness, and anger, which directly affect work capacity and concentration in daily activities and reduce the energy capacity to perform a given activity, greatly affecting HRQoL. In addition, the number of patients with epistaxis and/or wet purpura (mucosal bleeding) during diagnosis increased by one, the impact of ITP on patients’ HRQoL increased by 0.191 (*β* = 0.191, 95% CI: 0.091–4.259, *p* = 0.001), and the number of patients with clinical presentations of skin symptoms (petechiae and ecchymosis) of ITP patients increased by 0.041 (*β* = 0.041, 95% CI: 0.091–4.259 *p* = 0.041), which is in line with other studies conducted in China and Serbia (7, 27).

A study conducted in the United States of America (43) and China (40) showed that fatigue was one of the most debilitating aspects of the HRQoL of ITP. Moreover, in this study, the number of patients with fatigue during assessment also increased by one, and the impact of ITP on patients’ HRQoL increased by 0.236 (*β* = 0.326, 95% CI: 4.394–9.475, *p* < 0.001). The number of patients who did not take CPT increased by one, and the impact of ITP on patients’ HRQoL increased by 0.236 (*β* = 0.236, 95% CI: 2.236–6.570, *p* < 0.001). This may be because immunosuppressive therapy, especially high-dose corticosteroids, predisposes patients to infections, which may also affect HRQoL in ITP patients. A study conducted in China found that patients with primary ITP had a 24% incidence of infections in the first month of treatment, possibly due to immunosuppressive therapy. The number of patients living far from the clinic of hematology (out of Addis Ababa) increased by one, and the impact of ITP on patients’ HRQoL increased by 0.166 (*β* = 0166, 95% CI: 1.107–5.114 *p* = 0.003), which is in line with a study conducted in Serbia (7).

This study evaluated the impact of ITP on HRQoL in the Ethiopian population. In addition, the study assessed the impact of corticosteroid side effects on the HRQoL of ITP patients. The authors recommended that in this study the use of corticosteroids was not limited and that it strongly affected HRQoL domains. To reduce corticosteroid-related side effects, prednisone should be rapidly tapered and usually discontinued in responders, and in non-responders, medication should be discontinued after 4 weeks of initiation.

## 5 Limitation

Finally, this study had certain limitations. The maximum period (4 months) was used to recruit the study participants, but recruiting participants with rare diseases was challenging therefore, the sample size was small, and it is difficult to generalize the whole population. As this was a cross-sectional design, it was not possible to establish causal relationships or assess changes in HRQoL over time. Only patients who visited the hospital during the study period were included in the study. Those who were unable to attend the clinic may have had different experiences with their HRQoL.

## 6 Conclusion

The impact of ITP on their energy levels and work life was high as compared with the impact of ITP on daily life. The study on HRQoL domains and predictive factors for increasing impact of ITP on their HRQoL was the development of emotionally related corticosteroid side effects, presence of fatigue during the assessment, not taking CPT, living far from the hematology clinic (outside Addis Ababa), epistaxis and/or wet purpura (mucosal bleeding), and skin symptoms (petechiae and ecchymosis) during diagnosis. The side effects of corticosteroids also affect the HRQoL of ITP patients. In general, concerted efforts must be made to reduce the impact of ITP on HRQoL and prevent/manage corticosteroid side effects.

### 6.1 Ethical consideration

Ethical approval for the study and study protocol was obtained from AAU, CHS, School of Pharmacy ethical review board (approval number: ERB/SOP/487/14/2022). Before data collection, a written permission letter was obtained from the hematology/oncology unit of TASH and SPHMMC. The aims of the study were clearly explained to the study participants. The information was collected after obtaining written informed consent from each participant and taken from participants’ families/legal guardians for participants whose ages were between 14 and 18 years. The right was given to the study participants to refuse or discontinue participation at any time they wanted and the chance to ask anything about the study. For obscurity, the participant’s name was not used at the time of data collection, all other personnel information was kept entirely obscure, and confidentiality was assured throughout the study period.

## Data Availability

The original contributions presented in the study are included in the article/supplementary material, further inquiries can be directed to the corresponding author.

## References

[1] Marini I Zlamal J Pelzel L Bethge W Faul C Holzer U. Autoantibody-mediated desialylation impairs human thrombopoiesis and platelet lifespan. Haematologica. (2019) 134:2346. doi: 10.1182/blood-2019-131725 PMC7776251 31857361

[2] Omar IM Abuelela S Emam N. Value of pre-and post-treatment platelet indices in patients with immune thrombocytopenic Purpura. J Biosci Med. (2018) 6:11–24. doi: 10.4236/jbm.2018.69002

[3] Grozovsky R Hoffmeister KM Falet H. Novel clearance mechanisms of platelets. Curr Opin Hematol. (2010) 17:585–9. doi: 10.1097/MOH.0b013e32833e7561, PMID: 20729731 PMC4303238

[4] Rodeghiero F Stasi R Gernsheimer T Michel M Provan D Arnold DM. Standardization of terminology, definitions and outcome criteria in immune thrombocytopenic purpura of adults and children: report from an international working group. Blood. (2009) 113:2386–93. doi: 10.1182/blood-2008-07-162503 19005182

[5] Ozelo MC Colella MP de Paula EV do Nascimento ACKV Villaça PR Bernardo WM. Guideline on immune thrombocytopenia in adults: associação Brasileira de hematologia, hemoterapia e terapia celular. Project guidelines: associação médica Brasileira–2018. Hematol Transfus Cell Ther. (2018) 40:50–74. doi: 10.1016/j.htct.2017.11.001, PMID: 30057974 PMC6001928

[6] Cooper N Kruse A Kruse C Watson S Morgan M Provan D. Immune thrombocytopenia (ITP) world impact survey (iWISh): patient and physician perceptions of diagnosis, signs and symptoms, and treatment. Am J Hematol. (2021) 96:188–98. doi: 10.1002/ajh.26045 33170956 PMC7898610

[7] Suvajdzic N Zivkovic R Djunic I Vidovic A Markovic O Marisavljevic D. Health-related quality of life in adult patients with chronic immune thrombocytopenia in Serbia. Platelets. (2014) 25:467–9. doi: 10.3109/09537104.2013.831065, PMID: 24175579

[8] Efficace F Mandelli F Fazi P Santoro C Gaidano G Cottone F. Health-related quality of life and burden of fatigue in patients with primary immune thrombocytopenia by phase of disease. Am J Hematol. (2016) 91:995–1001. doi: 10.1002/ajh.24463, PMID: 27351715

[9] McMillan R Bussel JB George JN Lalla D Nichol JL. Self-reported health-related quality of life in adults with chronic immune thrombocytopenic purpura. Am J Hematol. (2008) 83:150–4. doi: 10.1002/ajh.20992, PMID: 17722072

[10] Snyder CF Mathias SD Cella D Isitt JJ Wu AW Young J. Health-related quality of life of immune thrombocytopenic purpura patients: results from a web-based survey. Curr Med Res Opin. (2008) 24:2767–76. doi: 10.1185/03007990802377461, PMID: 18715526

[11] Michel M Suzan F Adoue D Bordessoule D Marolleau JP Viallard JF. Management of immune thrombocytopenia in adults: a population-based analysis of the French hospital discharge database from 2009 to 2012. Br J Haematol. (2015) 170:218–22. doi: 10.1111/bjh.13415, PMID: 25824587

[12] Sestøl HG Trangbæk SM Bussel JB Frederiksen H. Health-related quality of life in adult primary immune thrombocytopenia. Expert Rev Hematol. (2018) 11:975–85. doi: 10.1080/17474086.2018.1548930 30444433

[13] Neunert C Terrell DR Arnold DM Buchanan G Cines DB Cooper N. American Society of Hematology 2019 guidelines for immune thrombocytopenia. Blood Adv. (2019) 3:3829–66. doi: 10.1182/bloodadvances.2019000966, PMID: 31794604 PMC6963252

[14] Kwiatkowska A Radkowiak D Wysocki M Torbicz G Gajewska N Lasek A. Prognostic factors for immune thrombocytopenic purpura remission after laparoscopic splenectomy: a cohort study. Medicina. (2019) 55:112. doi: 10.3390/medicina55040112, PMID: 31003557 PMC6524013

[15] Chugh S Darvish-Kazem S Lim W Crowther MA Ghanima W Wang G. Rituximab plus standard of care for treatment of primary immune thrombocytopenia: a systematic review and meta-analysis. Lancet Haematol. (2015) 2:e75–81. doi: 10.1016/S2352-3026(15)00003-4, PMID: 26687612

[16] Wang J Li Y Wang C Zhang Y Gao C Lang H. Efficacy and safety of the combination treatment of rituximab and dexamethasone for adults with primary immune thrombocytopenia (ITP): a meta-analysis. Biomed Res Int. (2018) 2018:1–12. doi: 10.1155/2018/1316096, PMID: 30648105 PMC6311778

[17] Trotter P Hill QA. Immune thrombocytopenia: improving quality of life and patient outcomes. Patient Relat Outcome Meas. (2018) 9:369–84. doi: 10.2147/PROM.S140932, PMID: 30568522 PMC6267629

[18] Grainger JD Young NL Blanchette VS Klaassen RJ. Quality of life in immune thrombocytopenia following treatment. Arch Dis Child. (2013) 98:895–7. doi: 10.1136/archdischild-2013-303784 23956257

[19] Vecchio R Intagliata E. Idiopathic thrombocytopenic purpura: current therapeutical strategies and review of the literature on outcome after splenectomy. Ann Laparosc Endosc Surg. (2020) 7:7. doi: 10.21037/ales-19-260

[20] Provan D Arnold DM Bussel JB Chong BH Cooper N Gernsheimer T. Updated international consensus report on the investigation and management of primary immune thrombocytopenia. Blood Adv. (2019) 3:3780–817. doi: 10.1182/bloodadvances.2019000812, PMID: 31770441 PMC6880896

[21] Irving G Neves AL Dambha-Miller H Oishi A Tagashira H Verho A. International variations in primary care physician consultation time: a systematic review of 67 countries. BMJ Open. (2017) 7:e017902. doi: 10.1136/bmjopen-2017-017902, PMID: 29118053 PMC5695512

[22] World Health Organization. World health statistics. Geneva: World Health Organization (2020).

[23] Terrell DR Neunert CE Cooper N Heitink-Pollé KM Kruse C Imbach P. Immune thrombocytopenia (ITP): current limitations in patient management. Medicina. (2020) 56:667. doi: 10.3390/medicina56120667, PMID: 33266286 PMC7761470

[24] Guidry JA George JN Vesely SK Kennison SM Terrell DR. Corticosteroid side-effects and risk for bleeding in immune thrombocytopenic purpura: patient and hematologist perspectives. Eur J Haematol. (2009) 83:175–82. doi: 10.1111/j.1600-0609.2009.01265.x, PMID: 19374704

[25] Brown TM Horblyuk RV Grotzinger KM Matzdorff AC Pashos CL. Patient-reported treatment burden of chronic immune thrombocytopenia therapies. BMC Blood Disord. (2012) 12:1–8. doi: 10.1186/1471-2326-12-2 22436142 PMC3350461

[26] Mathias SD Gao SK Miller KL Cella D Snyder C Turner R. Impact of chronic immune thrombocytopenic Purpura (ITP) on health-related quality of life: a conceptual model starting with the patient perspective. Health Qual Life Outcomes. (2008) 6:13–4. doi: 10.1186/1477-7525-6-13 18261217 PMC2275726

[27] Zhou Z Yang L Chen Z Chen X Guo Y Wang X. Health-related quality of life measured by the short form 36 in immune thrombocytopenic purpura: a cross-sectional survey in China. Eur J Haematol. (2007) 78:518–23. doi: 10.1111/j.1600-0609.2007.00844.x 17419740

[28] George JN Mathias SD Go RS Guo M Henry DH Lyons R. Improved quality of life for romiplostim-treated patients with chronic immune thrombocytopenic purpura: results from two randomized, placebo-controlled trials. Br J Haematol. (2009) 144:409–15. doi: 10.1111/j.1365-2141.2008.07464.x, PMID: 19016720

[29] Mathias SD Gao SK Rutstein M Snyder CF Wu AW Cella D. Evaluating clinically meaningful change on the ITP-PAQ: preliminary estimates of minimal important differences. Curr Med Res Opin. (2009) 25:375–83. doi: 10.1185/03007990802634119, PMID: 19192982

[30] Viana R D’Alessio D Grant L Cooper N Arnold D Morgan M. Psychometric evaluation of ITP life quality index (ILQI) in a global survey of patients with immune thrombocytopenia. Adv Ther. (2021) 38:5791–808. doi: 10.1007/s12325-021-01934-0, PMID: 34704193 PMC8572218

[31] Beyene DA Sisay EA Fentie AM Gebremedhin A. Reliability and validity of the Amharic version of immune thrombocytopenia life quality index tool for assessment of the health-related quality of life in Ethiopian patients of immune thrombocytopenia: cross-sectional study. SAGE Open Med. (2023) 11:20503121231199869. doi: 10.1177/20503121231199869, PMID: 37719164 PMC10504833

[32] Matzdorff A Meyer O Ostermann H Kiefel V Eberl W Kühne T. Immune thrombocytopenia-current diagnostics and therapy: recommendations of a joint working group of DGHO, ÖGHO, SGH, GPOH, and DGTI. Oncol Res Treat. (2018) 41:1–30. doi: 10.1159/000492187 30235458

[33] Cooper N Kruse A Kruse C Watson S Morgan M Provan D. Immune thrombocytopenia (ITP) world impact survey (I-WISh): impact of ITP on health-related quality of life. Am J Hematol. (2021) 96:199–207. doi: 10.1002/ajh.26036, PMID: 33107998 PMC7898815

[34] Jaime-Pérez JC Aguilar-Calderón P Jiménez-Castillo RA Ramos-Dávila EM Salazar-Cavazos L Gómez-Almaguer D. Treatment outcomes and chronicity predictors for primary immune thrombocytopenia: 10-year data from an academic center. Ann Hematol. (2020) 99:2513–20. doi: 10.1007/s00277-020-04257-2, PMID: 32945941

[35] Palau J Sancho E Herrera M Sánchez S Mingot ME Upegui RI. Characteristics and management of primary and other immune thrombocytopenias: Spanish registry study. Hematology. (2017) 22:1–9. doi: 10.1080/10245332.2017.1311442 28415913

[36] Grimaldi-Bensouda L Nordon C Michel M Viallard J-F Adoue D Magy-Bertrand N. Immune thrombocytopenia in adults: a prospective cohort study of clinical features and predictors of outcome. Haematologica. (2016) 101:1039–45. doi: 10.3324/haematol.2016.146373 27229715 PMC5060020

[37] Pamuk G Pamuk Ö Başlar Z Öngören Ş Soysal T Ferhanoğlu B. Overview of 321 patients with idiopathic thrombocytopenic purpura: retrospective analysis of the clinical features and response to therapy. Ann Hematol. (2002) 81:436–40. doi: 10.1007/s00277-002-0488-x, PMID: 12224000

[38] Provan D Stasi R Newland AC Blanchette VS Bolton-Maggs P Bussel JB. International consensus report on the investigation and management of primary immune thrombocytopenia. Blood. (2010) 115:168–86. doi: 10.1182/blood-2009-06-225565 19846889

[39] Rovó A Cantoni N Samii K Rüfer A Koenen G Ivic S. Real-world impact of primary immune thrombocytopenia and treatment with thrombopoietin receptor agonists on quality of life based on patient-reported experience: results from a questionnaire conducted in Switzerland, Austria, and Belgium. PLoS One. (2022) 17:e0267342. doi: 10.1371/journal.pone.0267342, PMID: 35446925 PMC9022837

[40] Yang R Yao H Lin L Ji J-m Shen Q. Health-related quality of life and burden of fatigue in Chinese patients with immune thrombocytopenia: a cross-sectional study. Indian J Hematol Blood Transfus. (2020) 36:104–11. doi: 10.1007/s12288-019-01124-7 32158092 PMC7042440

[41] Chakrabarti P George B Shanmukhaiah C Sharma LM Udupi S Ghanima W. How do patients and physicians perceive immune thrombocytopenia (ITP) as a disease? Results from Indian analysis of ITP world impact survey (I-WISh). J Patient Rep Outcomes. (2022) 6:1–14. doi: 10.1186/s41687-022-00429-y 35303181 PMC8933602

[42] Cooper N Cuker A Bonner N Ghanima W Provan D Morgan M. Qualitative study to support the content validity of the immune thrombocytopenia (ITP) life quality index (ILQI). Br J Haematol. (2021) 194:759–66. doi: 10.1111/bjh.17694, PMID: 34263940

[43] Grace RF Klaassen RJ Shimano KA Lambert MP Grimes A Bussel JB. Fatigue in children and adolescents with immune thrombocytopenia. Br J Haematol. (2020) 191:98–106. doi: 10.1111/bjh.16751, PMID: 32501532

